# Barriers to entering race training before 4 years of age for Thoroughbred horses born in the 2014 Australian foal crop

**DOI:** 10.1371/journal.pone.0237003

**Published:** 2020-08-05

**Authors:** Meredith L. Flash, Adelene S. M. Wong, Mark A. Stevenson, James R. Gilkerson

**Affiliations:** 1 Asia-Pacific Centre for Animal Health, Faculty of Veterinary and Agricultural Sciences, The University of Melbourne, Parkville, Victoria, Australia; 2 Equine Centre, Melbourne Veterinary School, Faculty of Veterinary and Agricultural Sciences, University of Melbourne, Werribee, Victoria, Australia; Massey University, NEW ZEALAND

## Abstract

Currently, there is a paucity of data on the barriers for Australian Thoroughbred horses transitioning from stud farm to racetrack. This paper reports the reasons why horses failed to enter race training and documents their exit destinations. Biographical records of Australian Thoroughbred horses born in 2014 were investigated to determine the number of horses that had not officially entered race training by the start of the 4-year old racing season (1 August 2018). Of the 13,677 foals born in 2014, 66% had commenced training and 51% had raced before the beginning of their 4-year-old season in Australia. A sampling frame based on the post code of the premises where foals were born and records from Racing Australia were used to select a geographically representative sample of the 2014 Australian Thoroughbred foal crop (*n* = 4,124). From the population eligible for sampling 1,275 horses that had not entered training were enrolled in the survey and their breeders were sent an online questionnaire with follow-up phone calls for those who had not responded. Of the 633 responses (50% of 1275) the most frequent outcomes for horses were: death (38%, *n* = 239), participation in the racing industry in their 4-year old racing season (24%, *n* = 154) and retirement (16%, *n* = 100) either as Australian Stud Book (ASB) bloodstock (*n* = 17), or as horses rehomed outside the Thoroughbred industry (*n* = 83). Illness or injury was the most frequent reason for horses not entering race training that were ASB bloodstock, rehomed or deceased. There was a loss of traceability at the point of sale with most horses sold at 1 year of age. This study provides important information on the reasons, alternative outcomes and gaps in traceability for horses not entering training prior to the 4-year-old racing season.

## Introduction

Thoroughbred racing and breeding are important contributors to the Australian economy and racing has long been considered an important part of the fabric of Australian society. A recent study estimated that the Thoroughbred industry (including breeding, racing businesses, and wagering) contributed more than AUD six billion to the Australian economy in 2016–2017 [1]. However, increasing scrutiny of perceived poor welfare standards in the Thoroughbred industry have the potential to threaten the social license under which the industry operates [2]. In particular, there has been concern over the number of horses that do not start racing, the reasons for this non-progression from the stud farm to the racetrack, and the outcomes of horses once they leave the Thoroughbred racing industry. To determine more precisely why foals do not enter racing it is important to define the population of interest, namely those Thoroughbred foals that do not start in a race. Previous studies investigating patterns of loss in the racing industry have included pre-foaling losses when mares fail to conceive or do not carry the pregnancy to term, as well as post-foaling losses such as illness and mortality in foals, and injuries associated with training and racing [3–5]. While pre-foaling losses are important, they are primarily a concern for the breeding industry. Post-foaling losses that occur prior to entering race training limit the number of horses available to participate in racing which is of concern to both the racing industry and the broader community.

The burden of disease is an important consideration when investigating the proportion of foals that progress from the stud farm to the racetrack. Previous studies on equine morbidity and mortality rates report considerable variation depending on the age group of horses studied and differences in the management practices between different horse facilities [6–9]. While there are mortality data published for other farmed animal species in Australia, such as sheep and cattle [10–12], it is not appropriate to compare these reported mortality rates with Thoroughbreds due to the inherently different systems of management. Compared to cattle and sheep breeding operations which manage their animals extensively, Thoroughbred broodmares in Australia are intensively and individually managed in protected yards separate from other animals during the peri-natal period. Previous equine studies in Australia have not investigated outcomes other than mortality of horses that fail to enter into training [5].

Losses that occur prior to commencement of race training are difficult to investigate, as there is often limited information about these horses recorded in official industry records. In Australia, the different state and territory Thoroughbred regulatory bodies had separate regulatory requirements prior to 2016. These requirements, along with the racing and breeding industries maintaining separate databases, contributed to a lack of a consistent population demographic data. The current lack of information on horses that fail to enter a training stable has resulted in negative speculation in the community regarding the outcomes of horses that fail to start a racing career. It is therefore important for the Thoroughbred industry to determine the outcome destinations for horses that fail to enter race training and the reasons for premature exit from the Thoroughbred industry. This will allow the industry to: (1) ensure that accurate information regarding outcomes and reasons for horses that do not enter training can be disseminated to the public; and (2) identify ways to increase the number of horses that are able to enter training. Accurate and open communication with the public regarding issues surrounding the welfare of horses will be beneficial for the industry. Policy development and discussions regarding racing’s social license will be better facilitated when both the general public and racing authorities are well-informed with accurate data.

The aim of this study was to investigate the reasons Australian Thoroughbreds failed to enter race training prior to four year of age, the alternative outcomes for those horses and the ages at which they had those outcomes. We selected a representative sample of Thoroughbred horses from the 2014 Australian foal crop that failed to officially enter a licensed training facility in Australia. The breeders of these horses were surveyed to determine the outcome of these untrained horses and the reasons they did not entering training.

## Materials and methods

The population of interest for this study were those horses from the 2014 Australian foal crop that had not officially entered race training within Australia by the start of their 4-year-old racing season (1 August 2018). Records were provided by the Australian Stud Book for all Thoroughbred foals born in Australia in the 2014 breeding season. These records included the postcode of the premises where the foal was born, which allowed the population to be assessed based on geographic distribution of mares at the time of foaling.

A combination of Australian Stud Book and Racing Australia records were used to link foals born in the 2014 season to the highest level of training and racing achieved prior to 1 August 2018 to identify those horses that had not entered race training prior to four years of age.

### Sampling

Sample size calculations were carried out to determine the number of Thoroughbred foals born in 2014 to be included in the study. We assumed that, as of 31 July 2018 each member of the 2014 foal crop were in one of four mutually exclusive outcome states: (1) alive and active within the Thoroughbred racing industry; (2) alive and active outside of the Thoroughbred racing industry (for example, as a pleasure horse); (3) exported; or (4) deceased. Horses in the first outcome state can be further categorised as racing, training (in preparation for racing) or waiting to enter training.

The proportion of 2014 foal crop horses that were alive and active within the Thoroughbred racing industry on 31 July 2018 was defined as *P*_1_, the proportion of horses that were alive and active outside of the Thoroughbred racing industry as *P*_2_, the proportion of horses that were exported as *P*_3_, and the proportion of horses that had died as *P*_4_. Here the assumption was that $\sum_{i=1}^{4}P_{i}=1$. Sample size calculations were carried out using the method of Tortora [13]. We assumed that *P*_1_…*P*_4_ took the values 0.50, 0.40, 0.05 and 0.05 (respectively) and we wanted to sample a sufficient number of 2014-born horses to be 95% certain that our estimates of the proportion of horses in each state on 31 July 2018 were within 5% of the true population values. Based on these assumptions, outcome details for a total of 2,600 horses born in 2014 across Australia were necessary to meet the requirements of the study.

Several additional assumptions were made in determining the final number of foals to be included in the study. It was assumed that outcomes of interest were not independent (i.e., that outcome states for individual horses were likely to be similar within breeding operations and within owners, compared to between owners and between studs). To account for this lack of independence in the data, the crude sample size estimate of 2,600 was adjusted accordingly. We assumed a conservative intra-class correlation coefficient of 0.05 and that, on average, enquiries would be made concerning three horses from each stud. This returned a design effect of 1.1, so the crude sample size estimate was multiplied by this value to return an adjusted sample size of 2,600 × 1.1 = 2,860 horses. We then assumed the non-response rate to the survey would be in the order of 30%, returning a minimum sample size of 2,860 ÷ (1–0.30) = 4,086 horses.

**Fig 1:**
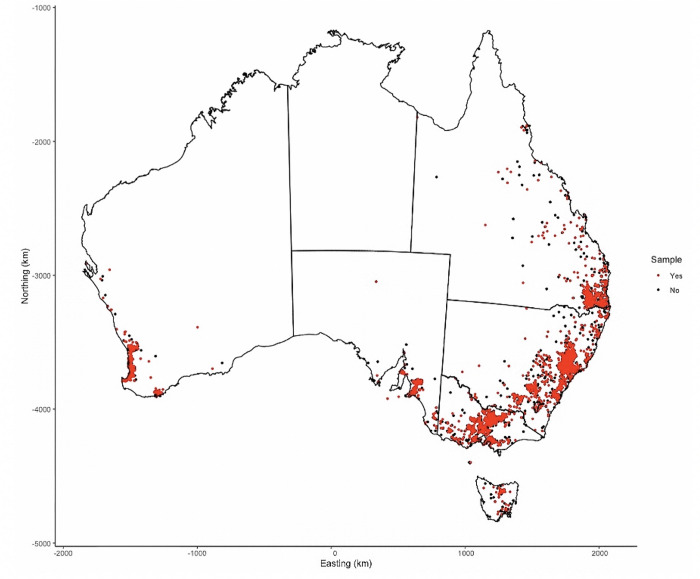
Distribution of the registered place of birth of the 2014 Australian foal crop. Map of Australia showing the location of sampled (red dot ●) and unsampled (black dot ●) Thoroughbred stud farms. Red dots are stacked on top of black dots where multiple stud farms exist in a single post code. Derived from Australian Bureau of Statistics [15] under a CC BY 4.0 license, with permission from Australian Bureau of Statistics, original copyright 2016.

A geospatial sampling frame was developed using the postcode of the stud farm where each foal was born. Longitude and latitude coordinates for each stud farm were randomly assigned so that the coordinates of each stud farm lay within the boundaries of its respective post code. Generalized Random Tessellation Sampling (GRTS) methods were used to select stud farms for inclusion into the study using a selection proportional to size approach to ensure the number of horses selected per stud farm in each state reflected the geographic distribution of the 2014 foal crop under investigation [14]. The location of the source population of stud farms and the stud farms selected for inclusion into the study are shown in Fig 1.

**Fig 2:**
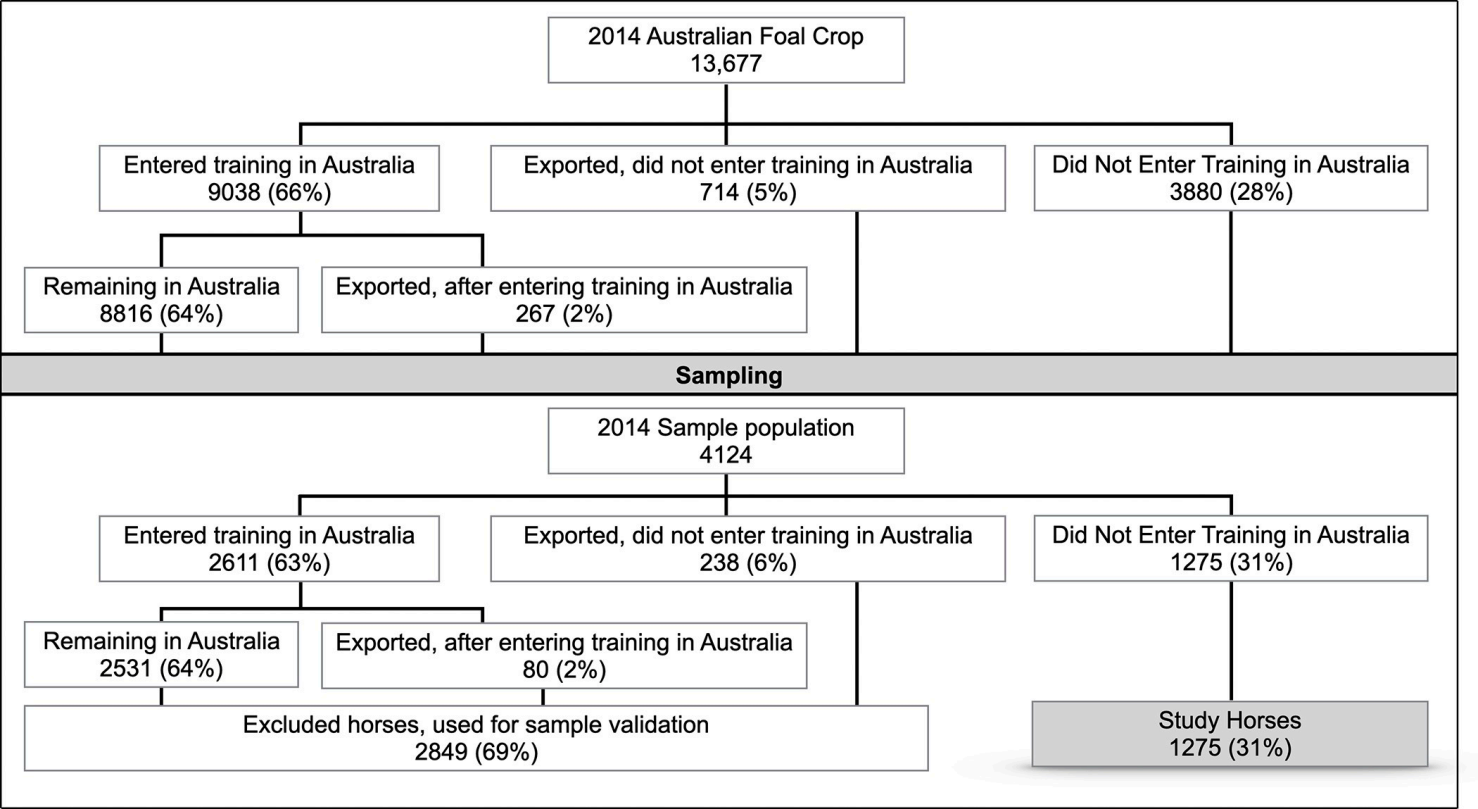
Flow chart of the highest level of training within Australia achieved for the 2014 Australian foal crop and sample population prior to the start of the 4-year-old racing season.

Of the 13,677 foals born in Australia in 2014, 365 had no foaling postcode recorded, and for the purpose of sampling, the contact postcode of the registered breeder was used instead. The proportion of horses born by state that were selected for sampling was compared to the numbers of foals born per state in 2014 to confirm that the sample was representative of the 2014 national foal crop. Racing and training records from Racing Australia were used to determine the highest level of training and racing for the horses selected for sampling. Horses in the study cohort were considered to have officially entered training if they had a stable return lodged, had participated in an official trial, or started in a race. A stable return is a document that records the presence of a horse in licensed premises, such as a training stable, and is required to be lodged with Racing Australia when a horse enters or leaves a registered trainer’s stable in Australia. An official trial is a practice race run on an official racetrack under the supervision of racing stewards. These records were compared to the equivalent data for the 2014 Australian foal crop to again confirm that the proportion of sampled horses that entered training and/or raced were consistent with the 2014 national foal crop population (Fig 2).

Within the group of horses selected for sampling (*n* = 4124), those with an official record of entering training in Australia or those where a training record was unable to be verified due to export were excluded. This left a cohort of 1,275 horses that had not entered training in Australia eligible for enrolment into the study (Fig 2). Further detail on the source population are provided in Supplementary Item 1.

### Questionnaire

Online questionnaires using Australian Stud Book biographical details were developed for each horse eligible for enrolment into the survey. Contact details for each breeder were provided by the Australian Stud Book. A personalised email was sent to each breeder, as the most current available registered industry contact, providing a hyperlink to the online questionnaire webpage for each of their eligible horse(s). The survey asked the respondent to nominate the age of the horse at the time of outcome, the outcome destination and reason for the outcome for horses that had no official record of entering training prior to 1 August 2018. Respondents were given the option of selecting the following outcome destinations: (1) actively racing or training for racing; (2) active non-stable training; (3) spelling; (4) Australian Stud Book mare or stallion; (5) sold at a public or private sale; (6) exported; (7) rehomed or retired; (8) deceased; and (9) other. Active non-stable training is a type of training that occurs outside of trainer’s licensed premise, such as pre-training or exercise at a water-walker. Options to select training and racing and active non-stable training were provided in the questionnaire to identify horses that entered race training for the first time as four-year-olds and to identify any gaps in the recording of horses training in industry records. Depending on the outcome destination provided for each horse, respondents were asked to provide additional details such as age at the time of outcome and reason for outcome as detailed in the decision tree in Supplementary Item 2. For example, if the respondent selected deceased as the outcome for a horse, they were asked to provide details on the circumstances, age and cause of death. If the cause of death was illness or injury, they were asked to provide information regarding the specific type of illness or injury.

Questionnaires were sent out to 965 breeders around Australia using contact information provided by the Australian Stud Book. Respondents were requested to provide information for the 1,275 unraced and untrained horses that were selected into the survey population. Where a breeder’s contact e-mail address was unavailable for a horse selected for the survey, another horse (*n* = 96) with a similar postcode (± one number) and category (unraced with no record of entering training) was substituted from the original national foal crop population.

Hyperlinks to the on-line questionnaire (S3 File) were sent out to breeders by e-mail on 24 December 2018. Every participant was sent an e-mail and subsequently followed up with a phone call if they had not completed their survey by 31 January 2019. The survey was closed for participants on 26 March 2019. Ethics approval for the study was granted by the University of Melbourne Human Research Ethics Committee (Approval number 1748714.1).

### Analysis of survey results

Analysis of survey results was carried out using Stata/IC Version 15.1. Categorical variables were compared using chi-squared tests where appropriate. The questionnaire (S2 File) asked respondents to describe the current status for the horse of interest. The categories for the current status included ‘other’ as an option where respondents felt that the listed options on the questionnaire were not appropriate. Respondents were asked to provide additional free-text comments about the current status if they selected ‘other’. Responses were manually reviewed and a number of responses in the ‘other’ category were re-classified into more specific categories based on the comments made by respondents that provided detail on the current status of their horse. Where re-classification was not possible the response was left as ‘other’. All results described in this report reflect the adjusted data incorporating these re-categorisations.

Horses categorised as (1) actively racing or training for racing and (2) active non-stable training were collapsed into a single category of ‘actively training/racing’ for analysis. Outcomes were cross checked for validity with industry records, such as in the case of surveys returns that categorised female horses as either ASB mare or broodmare for non-Thoroughbreds, to determine if they had a breeding record in ASB. The industry records for surveys that categorised horses as actively training/racing or spelling were compared to survey responses and are reported in the results. Data from Racing Australia (actively racing/training) for horses that began officially training and racing on or after 1 August 2018 were used to provide answers for a small number of respondents (32 of 633).

The outcomes for this study are reported as counts and percentages for all survey responses and each outcome category where appropriate. Confidence intervals for proportions were calculated using the Wilson Score method [16].

The survey sample of 1,275 horses was considered to be representative of the target population of all unraced and untrained horses in the 2014 national foal crop. Sample outcome proportions and confidence intervals were used to calculate point estimates and ranges for the target population of 3,880 horses that comprised the proportion of the 2014 foal crop that had not entered the official training or racing record system prior to the start of their four-year-old racing season. Because this study provided an essentially random sample of unraced and untrained horses, there was no requirement to adjust our survey results to account for the sampling study design [17].

## Results

Responses were received from 509 breeders for 633 of the 1,275 horses enrolled in the survey, yielding a 50% (95% CI 47, 52) response rate. The median number of horses per respondent was one (Q1 [Quartile 1] 1; Q3 [Quartile 3] 1; Maximum 12). Responses were received for 328 female and 305 male horses. The percentage of survey responses received was similar across all states (χ^2^ test statistic 4.09; df = 6; p = 0.66).

Responses were not received for 642 horses from 447 breeders. These non-respondents could be divided into several categories. Thirty-nine percent of 447 non-responding breeders (*n* = 175 breeders; *n* = 207 horses) could not be contacted due to incorrect e-mail addresses, disconnected or incorrect phone numbers or they were deceased or were indisposed; 37% (*n* = 164 breeders; *n* = 250 horses) could not be reached with voice messages left on their phones; 11% (*n* = 51 breeders; *n* = 69 horses) could not be reached with no option to leave a message and 8% (*n* = 34 breeders; *n* = 80 horses) indicated they would respond later with more information but had not completed the survey by the closing date. Only 5% (*n* = 23 breeders, *n* = 36 horses) breeders selected for enrolment in the survey chose to opt out of the survey.

### Outcomes of surveyed horses

#### Participating in training or racing

Twenty four percent of horses (*n* = 154) were categorised as actively participating in training or racing activities, despite no record of this activity in an official racing database at the time of selection into the study.

Eighteen percent of horses, (*n* = 117) were categorised as actively participating in race training in Australia as a four-year-old at the time of the survey, and 6% (*n* = 37) were categorised as spelling, based on the assumption that a spelling horse must previously have undergone some form of training or pre-training (Table 1). These horses were retained in this study because they were not recorded as racing or training in official racing records prior to the beginning of their 4-year-old racing season (1 August 2018).

**Table 1:** Outcomes of surveyed horses.

Outcome	Number of horses	Percentage of total respondents (95% CI)
Deceased		
< 1 year old	125	20 (17, 23)
≥ 1 year old	114	18 (15, 21)
Participating in the racing industry		
Actively racing/training	117	18 (16, 22)
Spelling	37	6 (4, 8)
Public sale/private sale	84	13 (11, 16)
Rehomed/retired	83	13 (11, 16)
Australian Stud Book mare/stallion	17	3 (2, 4)
Exported	1	0.2 (0, 1)
Other–Intending to race	20	3 (2, 5)
Unknown	35	6 (4, 7)
Total	633	100

Racing Australia training and racing records from 1 August 2018 to 31 December 2018 were analysed for the 117 horses categorised as ‘actively racing or training’ and 37 horses categorised as ‘spelling’. For 41% (*n* = 48) of horses that were categorised as ‘actively racing or training’ and 8% (*n* = 3) of ‘spelling’, the horse had recorded a stable return, trialed or raced during the follow up period (1^st^ August-31^st^ December 2018). Collectively, these horses that officially entered training in the follow up period represent 8% (n = 51) of the total survey responses. The remaining 16% (*n* = 103) of horses categorised as participating in the racing industry had no official record in Racing Australia data of being in training, suggesting that any training activities were unofficial, and these horses did not start in a race event administered by Racing Australia.

Although the survey population was selected on the basis of having not entered training, a further 3% (*n* = 36) of horses were categorised as having been involved in some level of pretraining or training. Five percent (*n* = 4) of horses in the ‘public or private sale’ outcome category, 39% (*n* = 32) from the ‘rehomed/retired’ category, and 41% (*n* = 7) from the ‘Australian Stud Book mare/stallion’ category were reported as having undergone training or pretraining prior to their outcome. Respondents also reported that 8% (*n* = 19) of 239 horses categorised as ‘deceased’ were injured during training or pretraining.

Across all outcome categories, 34% (*n* = 216) of horses had undergone varying levels of unofficial training prior to the outcome or had subsequently entered training for the first time as 4-year-olds.

#### Deceased

The most frequent (38%, *n* = 239) reason identified by respondents for horses not participating in the racing industry was that the horse had died before it entered training. The period of greatest mortality risk was the period from birth to 12 months of age (52%, 125 of 239) (Table 1).

Of the 125 deaths that occurred before one year of age, 47% (*n* = 59) occurred during the neonatal period (less than one month of age), 38% (*n* = 48) occurred between one and six months of age and the remaining 14% (*n* = 18) occurred from six to 12 months of age. In the neonatal period, the most critical period was the first week with 76% of these foals (*n* = 45) dying within 7 days of birth.

Fracture was the most frequently reported cause of death overall (*n* = 44) and for horses one year of age and older (*n* = 33). The most frequently reported cause of death in horses less than one year of age was congenital malformation. There was no difference in the percentage of deceased horses whose cause of death was unknown (18%) for horses that died under one year of age (*n* = 22) compared with those (*n* = 21) that died at one year of age or older (χ^2^ test statistic 0.02; df = 1; p = 0.87).

Eight percent (*n* = 19) of 239 horses categorised as deceased were reported as having died during unofficial training or pretraining, without ever registering a stable return with Racing Australia. Five of these horses reported to have died at one year of age, seven at two years of age, and seven at three years of age.

Traumatic causes of mortality, including fracture, tendon/ligament injuries, wounds/trauma or horses that had multiple traumatic injuries were reported for 32% (*n* = 77) of deceased horses. This included horses where respondents selected multiple causes of death (Table 2). Nineteen percent (*n* = 15) of the 77 traumatic injuries such as fracture, tendon/ligament injury, multiple injuries and wounds/trauma, were reported to have occurred during training/pretraining, although there was no official record of these horses having entered training. The remaining 81% (*n* = 62) were due to paddock accidents or misadventure-type incidents.

**Table 2:** Age distribution and reasons for death of horses.

Reason for death	Number of horses				
	Neonatal period	≥ 1 month, < 1 year old	1 to 4 years old	Age not stated	Total
Fracture	4	7	31	2	44
Congenital malformation	14	8	7	1	30
Digestive condition	3	10	14	0	27
Sudden death	3	7	6	0	16
Infection	4	7	4	0	15
Wound/Trauma	2	6	7	0	15
Tendon/Ligament Injury	0	0	9	0	9
Complications at birth	8	0	0	0	8
Immune condition	5	0	1	0	6
Lower respiratory	1	2	2	0	5
Neurologic condition	1	1	2	0	4
Upper respiratory condition	0	3	0	0	3
Drowned	2	0	1	0	3
Cardiac/metabolic condition	1	1	0	0	2
Multiple conditions^†^	1	2	5	1	9
Unknown/Unspecified	10	12	9	12	43
**Total**	**59**	**66**	**98**	**16**	**239**

^†^ Multiple reasons of death could be selected by the survey participant. One horse was listed to have died from infection, tendon/ligament injury, and congenital malformation; three horses from fracture and tendon/ligament injury; three horses from infection and tendon/ligament injury; and two horses from fracture and wound/trauma.

Overall, 73% (*n* = 175) of deceased horses were reported to have died at < 2 years of age. These horses were not old enough to be eligible to start in a race at the time of their death.

Three horses categorised as deceased were reported to have either died *in utero* (*n* = 1) or were stillborn (*n* = 2) showing that there were horses recorded as part of the foal crop that were not actually live births.

#### Public or private sale and export

Thirteen percent (*n* = 84) of horses were categorised as having been sold at public or private sales (Table 1). The main reason for sale was cited as owner request/proactive decision (Table 3). The largest age group for horses being sold was the one-year-olds (48%, 40 of 84 horses), followed by horses less than one year of age (25%, 21 of 84 horses), 2 horses were sold *in utero*, 10 were between 2–4 years of age and the ages for 11 horses were not specified.

**Table 3:** Surveyed reasons for the sale of horses.

Reason for sale	Number of horses
Owner request/proactive decision	73
Poor performance/slow	2
Unsuitable temperament/behavior	2
Other	2
Not stated	5
Total	84

The majority (62%, *n* = 45) of the survey responses for the 73 horses where the reason for sale was ‘owner request/proactive decision’, identified that the horses were sold at weanling or yearling sales, and/or had been recorded on industry sale records. Respondents often indicated that they were not aware of the outcome of horses once they had been sold.

Less than one percent (*n* = 1) of horses were categorised as having been exported (Table 1). The age at export of this horse was not listed by the survey respondent.

#### Rehomed or retired

Thirteen percent (*n* = 83) of horses were categorised as having been rehomed or retired (Table 1) with 61% (*n* = 51) of them rehomed or retired prior to undergoing any form of unofficial training or pretraining.

The main reasons for being rehomed or retired were due to an illness or injury followed by poor performance (Table 4). The most common types of injuries were tendon/ligament injuries (*n* = 7) followed by congenital malformation (*n* = 6) and wounds or trauma related injuries (*n* = 5).

**Table 4:** Reasons for rehoming/retirement.

Reason for retirement	Number of horses	Number of horses unofficially training prior to outcome
Injury/Illness	27	9
Poor performance	22	17
Owner request/proactive decision	11	4
Personal/Financial reasons	8	-
Unsuitable temperament/behavior	5	1
Other	3	1
Not stated	7	-
Total	83	32

The detail provided by the survey respondents regarding the specific congenital malformation suggest that conformation fault and congenital malformation were used interchangeably, with ‘bad knee conformation’, ‘weak hocks’ and ‘born premature’ categorised as congenital malformation.

Fifty-three responses for retired or rehomed horses, reported the age of retirement. Of these responses the majority (42%, *n* = 22) reported the horse retired at 3 years of age, followed by 2 years of age (*n* = 12) and 4 years of age (*n* = 8). Horses were mainly rehomed into equestrian or pleasure pursuits followed by companion and other unridden activities (Table 5). For horses that were categorised as rehomed to equestrian or pleasure pursuits, pleasure horse or hack was the most frequent category (*n* = 25), followed by pony club (*n* = 8), adult riding (*n* = 3), dressage (*n* = 3) and show jumping (n *=* 3).

**Table 5:** Types of pursuits when retired or rehomed.

Pursuits	Number of horses
Equestrian and pleasure	42
Companion and other unridden activities	29
Broodmare for non-Thoroughbreds	1
Other	2
Not specified	9
Total	83

#### Australian Stud Book mare/stallion

Three percent (*n* = 17) of horses were categorised as being used for breeding as Australian Stud Book mares/stallions at the time of the survey (Table 1).

Like horses categorised as retired or rehomed, injury or illness was the most frequent reason for horses becoming Australian Stud Book mares/stallions (*n* = 8) with congenital malformation/poor conformation the most common specific reason. Conformation fault or congenital malformation was the most frequent specific illness or injury reported, with poor leg or hoof conformation reported for five of the eight horses.

The majority of the 17 horses were reported as becoming Australian Stud Book mares/stallions at the age of three (*n* = 9), followed by four years of age (*n* = 2) and two years of age (*n* = 1) with age not specified for five horses.

#### Other–intending to race

A further 3% (*n* = 20) of horses, were categorised by survey respondents as having a status other than the options listed in the survey and were reported as being held with an intent to race. Respondents indicated that these horses had not yet entered training because of financial or personal reasons (*n* = 6), they had wanted the horse to grow and mature (n = 2), or the horse had experienced an injury when it was young (*n* = 2). The remaining 10 respondents did not provide an outcome reason.

#### Unknown

The current status, at the time of the survey, of 6% (*n* = 35) of horses was reported as unknown Table 1). Respondents either indicated that they could not remember, did not know the outcome of the horse, or did not select an outcome.

#### Extrapolation of survey results to the 2014 Australia foal crop

Survey findings from the 633 responses to the survey questionnaire were extrapolated to provide estimates of the frequency of the major outcomes at the national population level for the 2014 foal crop. These findings relate to the estimated national population of 2014-born horses that had not entered training by the start of their four-year-old racing season. National records indicate that of 13,677 foals born in 2014 (the national foal crop), 3,880 (28%) had not entered training in Australia prior to the start of their four-year-old racing season (S1 File).

Table 6 provides estimates of counts and percentages of animals in each of the major outcome categories for the survey sample and for the proportion of the national 2014 foal crop population that did not enter training prior to their 4-year-old season (*n* = 3,880).

**Table 6:** Extrapolation of survey results to the total number of horses that had not registered an official stable return prior to 1 August 2018.

Outcome	Survey sample (n)	Survey sample (% and 95% CI)	Estimated count at the national 2014 population level (95% CI)
Deceased			
< 1 year old	125	20 (17, 23)	764 (652–892)
≥ 1 year old	114	18 (15, 21)	698 (590–823)
Participating in the racing industry			
Actively racing/training	117	19 (16, 22)	718 (609–842)
Spelling	37	6 (4, 8)	225 (167–310)
Public sale/private sale	84	13 (11, 16)	516 (423–625)
Rehomed/retired	83	13 (11, 16)	508 (415–621)
Australian Stud Book mare/stallion	17	3 (2, 4)	105 (66–167)
Exported	1	0.2 (0, 1)	6 (0–35)
Other–Intending to race	20	3 (2, 5)	124 (81–186)
Unknown	35	6 (4, 8)	213 (155–295)
**Total**	**633**	**100**	**3878**^**a**^

^a^ Subject to rounding error.

Extrapolation of survey results to the 3,880 horses that had not entered training or raced by the beginning of their 4-year-old season estimate the number of horses in the 2014 Australian foal crop that would have died in Australia prior to one year of age to be 764, or approximately 6% of the foal crop (Table 6, *n* = 13,677).

Extrapolation of the survey results across the Australian foal crop shows that there were 943 horses that did not have an official record of racing or training in Australia prior to their four-year-old racing season (1 August 2018), but were actively participating in the industry (Table 6). This estimated number makes up 7% of the 2014 Australian foal crop (*n* = 13,677) that would have either begun their official training after four years of age or had undergone training through unofficial means not captured by official records.

## Discussion

To the best of our knowledge this is the first study to provide estimates of the percentage of Australian Thoroughbreds from a foal crop that did not enter race training, providing insight into barriers for entering race training. The study was designed to ensure that the survey sample was representative of Australian Thoroughbred racehorses born in a single year.

Care should be taken when using the findings of this study when describing the number of horses in total that train and race from the 2014 Australian foal crop. The period of interest for this study was prior to and including the start of the 2018–2019 racing season. It is possible that some horses may have started training and racing after the follow up period, so the percentage of horses training and racing report in this study may, in fact, be an underestimate of the total number of horses entering training and racing from the 2014 foal crop. The proportion of horses entering training and racing after 4-years of age is likely to be small so the impact of this bias on our overall findings is reasoned to be low. There were also a number of horses in several other categories (‘deceased’, ‘sold at a public or private sale’, ‘rehomed or retired’ or ‘Australian Stud Book mare or stallion’) that were also reported by survey respondents as having undergone unofficial training or pretraining prior to their final status, despite having no record of a stable return in the racing database. This suggests that official Racing Australia records overestimate the proportion of horses that do not enter training. This overestimation is due to records of training on licensed premises not fully capturing the total number of horses that enter training, as some horses may be training at non-registered premises, or the paperwork to officially register the horses at licensed premises may not have been completed in a timely manner. Horses that were sold may have entered training however the lack of traceability from point of sale prevents investigation of horses that unofficially trained and any other outcomes after leaving the breeder’s care. The introduction of rules in 2016 (AR 34 and AR 50) that require horses to be recorded with Racing Australia within 30 days of birth should assist in improving traceability of horses from stud farm through point of sale through to race training [18].

Another important observation was that 8% of survey horses were officially recorded as racing or actively participating in race training after the age of four and a further 3% were reported by survey respondents as being held with an intention to train and race in the future. Explanations for the delayed start of training and racing included financial or personal reasons and concerns over whether the horse was ready for training or the breeder’s personal belief that starting training of horses at young ages was harmful. This observation from survey respondents is not consistent with the increasing body of evidence that the early introduction of exercise has a positive association with career length and racing success for Thoroughbred horses [19–22]. Age at first start, age when first trialled, and age first registered with a trainer have all been identified as having a positive correlation with career duration [19–23]. Further research is needed to better understand the financial and personal reasons preventing healthy, fit for purpose horses from entering training.

Although official industry records overestimated the proportion of the foal crop that did not enter training, the main reason for foals not progressing to become racehorses was death. The population of interest for this study includes all horses that died from the entire foal population up until about 12 months of age, so that extrapolated figures for horses that were deceased prior to one year of age provide a valid national estimate of risk. The incidence risk of death prior to one year of age was 6% in the national crop of foals born. The highest period for risk of mortality was less than 12 months of age with congenital malformation the most common cause of death in this age group. Survey responses appeared to use congenital malformation and conformation fault interchangeably which may have resulted in an overestimation of the frequency of congenital malformation for this study. Further information is needed on the type and incidence of congenital malformation and conformation faults to better understand the incidence of each condition, the risk of them occurring and their effect on horses’ transition from the stud to racetrack.

Deaths beyond 12 months of age were not able to be extrapolated accurately beyond the untrained and unraced horses of the 2014 national foal crop. These deaths provide an estimate of the relative frequency of different causes of death in these untrained and unraced horses but do not provide any meaningful population measure of mortality risk. As a whole, our survey results indicate that the majority of deaths that occurred were due to non-training related illnesses or injuries. There is currently no available research on stud farm design that addresses the potential for injuries to occur to horses on the stud farm. Further research is needed to investigate how stud farm design and infrastructure are associated with risk of fatality in horses.

The limitations of this study were the limitations common to many surveys. The most important of these was that answers given by survey respondents are subject to recall bias and reliant on the memory or good record keeping of survey respondents in order to answer the questions accurately. Recall bias may also have resulted in a loss of detailed information for horses where the outcome was in the first years of life for this cohort. To reduce the possibility of recall bias the respondents were provided with a relatively large amount of industry information about the individual horses of interest. The median number of horses per breeder was one (in agreement with the wider foal crop, S1 File) which further decreased the likelihood of poor recall.

## Conclusion

Mortality was the most frequent barrier preventing foals commence training, with the majority of these deaths occurring before these foals reached one year of age. A large proportion of foal deaths occurred in the first month of life. While official race records provide an accurate indication of the number of horses that start in a race, industry data underestimates the proportion of the national foal crop that enters training, with some horses beginning their training and racing career at four years of age.

## Supporting information

S1 File Analysis of source population. (DOCX)

S2 File Australian thoroughbred wellbeing survey. (DOCX)

S3 File Link to an example survey. (DOCX)

S4 File Dataset. (CSV)
