# Supplementary material for: Barriers to entering race training before 4 years of age for Thoroughbred horses born in the 2014 Australian foal crop
Source: PLoS One. 2020 Aug 5;15(8):e0237003. doi: 10.1371/journal.pone.0237003 (PMC7406052; doi:10.1371/journal.pone.0237003)
Supplement: S1 File — (DOCX) [file pone.0237003.s001.docx]

**Supplementary Item 1**

**Analysis of the source population**

Based on Australian Stud Book records, a total of 13,677 Thoroughbred foals were born in Australia in 2014. Population data were collected from a combination of Australian Stud Book and Racing Australia records up to 31 July 2018, the end of the 2017-2018 racing season. Industry records after that date were not considered during the selection of the survey sample.

From their birth until the end of the 2017-2018 racing season (31 July 2018), 66% of the 2014 foal crop were reported to have entered training, as they had registered at least one stable return and/or participated in an official trial and/or raced with Racing Australia. A total of 51% of the foal crop had recorded at least one start in a race, and 15% had trialled or registered a stable return but had not raced. Seven percent (*n* = 981) of the 2014 foal crop were recorded by the Australian Stud Book to have been exported from Australia. Two hundred and sixty-seven of these horses had already raced or trained in Australia prior to export, but 5% were exported prior to entering training in Australia. Of the horses remaining in Australia, 28% (*n* = 3880) had no official record of entering training.

The median number of horses per breeder was one (Q1 [Quartile 1] 1, Q3 [Quartile 3] 3, Max 201) across the 4488 breeders identified by the Australian Stud Book as having a foal born in Australia in 2014. The largest number of foals (*n* = 6124) were born in a single state in New South Wales (NSW). Foals born in NSW, Victoria and Queensland comprised 85% (*n* = 11,578) of the entire 2014 Thoroughbred foal crop (S1 Table 1). The first random, geospatial sampling step produced a sample of 4,124 foals, representing 30% of the total foal crop which was distributed across all post codes in Australia where mares foaled (Fig 2).

**Supplementary Table 1. Number of Thoroughbred foals born in each State and Territory of Australia in 2014**

| **State** | **Foals born per state** | **Percentage of foals (%)** |
| --- | --- | --- |
| New South Wales | 6,124 | 44.8 |
| Victoria | 3,328 | 24.3 |
| Western Australia | 1,305 | 9.5 |
| Queensland | 2,126 | 15.5 |
| South Australia | 524 | 3.8 |
| Tasmania | 251 | 1.8 |
| ACT | 18 | 0.1 |
| Northern Territory | 1 | 0.0 |
| Total | 13,677 | 99.8^†^ |

^†^ Subject to rounding error.

Of the 4,124 horses included in the sample population, 63% (*n* = 2611) had a record of entering training before 1 August 2018, and 6% were horses that were exported prior to entering training in Australia and these were therefore excluded from the survey (Fig 2). A total of 1,275 horses had no record of export or entering training in Australia. These animals then formed the survey sample for additional survey data collection (Fig 2).
