# Supplementary material for: Barriers to entering race training before 4 years of age for Thoroughbred horses born in the 2014 Australian foal crop
Source: PLoS One. 2020 Aug 5;15(8):e0237003. doi: 10.1371/journal.pone.0237003 (PMC7406052; doi:10.1371/journal.pone.0237003)
Supplement: S2 File — (DOCX) [file pone.0237003.s002.docx]

# Supplementary Item 2

**Australian Thoroughbred Wellbeing Survey**

**Horse information**

Records obtained from the Australian Stud Book and Racing Australia identify you as the registered breeder, owner, or trainer of:

Life number: (prefilled data)

Name: (prefilled data)

Sire: (prefilled data)

Dam: (prefilled data)

Sex: (prefilled data)

Colour: (prefilled data)

Date of Birth: (prefilled data)

To the best of your knowledge, is “horsename” (prefilled data) currently:

1. Actively racing/Race training (2018/2019 season)
2. Active non-stable training (i.e. pretraining, water walker, etc.) (2018/2019 season)
3. Spelling
4. Australian stud book mare/stallion
5. Rehomed/Retired
6. Livestock sale/private sale
7. Exported
8. Deceased
9. Other - please state in the box below

**DECISION TREE AND QUESTIONS**

**Options (B) and (C)**

Active non-stable training

Spelling

**Option (G)**

Exported

**Options (D) and (F)**

Australian Stud Book mare/stallion

Livestock sale/private sale

**Options (A) and (I)** Actively racing/Race training, Other

Other

1. At what age was the horse exported?
2. What country was the horse exported to?
3. FINAL COMMENTS (See page 5)
4. FINAL COMMENTS (See page 5)
5. If horse was not raced or trialed prior to the chosen outcome, had the horse undergone any training/pretraining?

Yes

No

N/A

1. At what age did the horse leave the industry?
2. Why did the horse leave the industry? Once you have selected an option, please provide us with any additional information in the comments box below. If you select “injury/illness” please proceed to the next page for more follow-up questions

Injury/Illness

Poor performance/slow

Unsuitable temperament/behaviour

Owner request/proactive decision

Other – please specify

3a. (If “injury/illness” was selected)

What was the injury or illness? Once you have selected an option please comment on the specific injury, if known, as well as any additional information you might have in the comments box below.

Fracture

Tendon/Ligament injury

Upper respiratory condition i.e. roarer, displaced soft palate, strangles etc.

Lower respiratory condition i.e. bleeder, asthma, pneumonia, etc.

Cardiac/metabolic condition i.e. arrhythmia, haemorrhage, etc.

Digestive condition i.e. colic, peritonitis, etc.

Congenital malformation i.e. contracted tendons, wobbler, etc.

Immune condition i.e. neonatal isoerythrolysis, etc.

Other (please specify)

1. FINAL COMMENTS (See page 5)
2. What date did the horse begin its spelling/non-stable training period?
3. What is the intended return to race training date if known?
4. If spelling, what is the reason for spelling?

Injury/Illness

Other (please specify)

3a. (If “injury/illness” was selected)

What was the injury or illness? Once you have selected an option please comment on the specific injury, if known, as well as any additional information you might have in the comments box below.

Fracture

Tendon/Ligament injury

Upper respiratory condition i.e. roarer, displaced soft palate, strangles, etc.

Lower respiratory condition i.e. bleeder, asthma, pneumonia, etc.

Cardiac/metabolic condition i.e. arrhythmia, haemorrhage, etc.

Digestive condition i.e. colic, peritonitis, etc.

Congenital malformation i.e. contracted tendons, wobbler, etc.

Immune condition i.e. neonatal isoerythrolysis, etc.

Other (please specify)

1. FINAL COMMENTS (See page 5)
2. Which of the following best describes when the death of this horse occurred? Once you have selected an option, please provide us with any additional information you might have in the comments box below.

Race

Trial/jumpout

Training/pretraining

Other (please specify)

1. What was the horse’s age of death?
2. What was the reason for death?

Injury/Illness

Sent to abattoir

Other (please specify)

3a. (If “injury/illness” was selected)

What was the injury or illness? Once you have selected an option please comment on the specific injury, if known, as well as any additional information you might have in the comments box below.

Fracture

Tendon/Ligament injury

Upper respiratory condition i.e. roarer, displaced soft palate, strangles etc.

Lower respiratory condition i.e. bleeder, asthma, pneumonia, etc.

Cardiac/metabolic condition i.e. arrhythmia, haemorrhage, etc.

Digestive condition i.e. colic, peritonitis, etc.

Congenital malformation i.e. contracted tendons, wobbler, etc.

Immune condition i.e. neonatal isoerythrolysis, etc.

Other (please specify)

OR

3b. (If “sent to abattoir” was selected)

What was the reason for sending to abattoir? Please provide us with further details, if known, in the comments box below.

Owners request

Behaviour

Injury/Illness

Performance

Other (please specify)

1. FINAL COMMENTS (See page 5)
2. Please check the box most applicable to the outcome of the horse. Please provide us with specifics/any additional information you might have in the additional comments box below.

Equestrian and pleasure pursuits: Dressage, show jumping, eventing, pony club, adult riding, trail riding, etc. If you have selected this option, please proceed to the next page for more follow-up questions.

Companion or other un-ridden activities – please state what these are in the box below

Broodmare for non-thoroughbreds

Re-homed within the racing industry as a lead pony or clerk of the course

Other – please state what these are in the box below

1. (If Equestrian and pleasure pursuits was selected)

Which options best describe the equestrian/pleasure pursuits applicable to the horse? Please use the additional comments box to provide any additional details you might have about the individual pursuits.

Dressage

Show jumping

Eventing

Pony club

Adult riding

Pleasure horse/Hack

Other – please state in the box below

1. If horse was not raced or trialed prior to the chosen outcome, had the horse undergone any training/pretraining?

Yes

No

N/A

1. At what age did the horse leave the industry?
2. Why did the horse leave the industry? Once you have selected an option, please provide us with any additional information in the comments box below. If you select “injury/illness” please proceed to the next page for more follow-up questions

Injury/Illness

Poor performance/slow

Unsuitable temperament/behaviour

Owner request/proactive decision

Other – please specify

5a. (If “injury/illness” was selected)

What was the injury or illness? Once you have selected an option please comment on the specific injury, if known, as well as any additional information you might have in the comments box below.

Fracture

Tendon/Ligament injury

Upper respiratory condition i.e. roarer, displaced soft palate, strangles etc.

Lower respiratory condition i.e. bleeder, asthma, pneumonia, etc.

Cardiac/metabolic condition i.e. arrhythmia, haemorrhage, etc.

Digestive condition i.e. colic, peritonitis, etc.

Congenital malformation i.e. contracted tendons, wobbler, etc.

Immune condition i.e. neonatal isoerythrolysis, etc.

Other (please specify)

1. FINAL COMMENTS (See page 5)

**Option (H)**

Deceased

**Option (E)**

Rehomed/Retired

**FINAL COMMENTS**

Do you have any further comments regarding the outcome/current state of the horse?

Would you be happy for us to contact you if we have any further questions?

**Yes**

**No**

******** END OF SURVEY ********
