# Supplementary material for: Barriers to entering race training before 4 years of age for Thoroughbred horses born in the 2014 Australian foal crop
Source: PLoS One. 2020 Aug 5;15(8):e0237003. doi: 10.1371/journal.pone.0237003 (PMC7406052; doi:10.1371/journal.pone.0237003)
Supplement: S3 File — (DOCX) [file pone.0237003.s003.docx]

**Supplementary Item 3**

**An example survey can be found here:** https://www.surveymonkey.com/r/TK2MS6Q?&n=Fast%20As&s=Quick%20Boots&d=Lightning%20McQueen&sx=Male&db=1/5/2016&cr=Pink%20Striped
